# Recurrent Achilles Tendon Rupture Following Open Surgical Repair, Treated Nonoperatively, and Enhanced With Biological Platelet-Rich Plasma (PRP) Therapy: A Case Report

**DOI:** 10.7759/cureus.81441

**Published:** 2025-03-29

**Authors:** Andrei Visoianu, Gabriela Soare, Constantin Cosmin Baciu, Razvan Ene

**Affiliations:** 1 Orthopedics and Traumatology, Faculty of Medicine, "Carol-Davila" University of Medicine and Pharmacy, Bucharest, ROU; 2 Orthopedics and Traumatology, Clinical Emergency Hospital, Bucharest, ROU

**Keywords:** achilles tendon, open repair, prp, rerupture, tendon recovery

## Abstract

Achilles tendon ruptures are common injuries, often occurring in active individuals, with surgical repair being a standard approach for complete ruptures. Cases of open re-rupture of the Achilles tendon represent a rare complication after open surgical repair of that tendon. Literature offers only one or two cases from every author without a consensus on how to deal with this pathology, so each patient should be treated according to the particularities of the case. This case report highlights the management and challenges associated with an open re-rupture of the Achilles tendon in a 35-year-old male patient following primary surgical repair. The patient, a smoker with a BMI of 28.6, initially presented with a complete mid-substance rupture sustained during recreational football. Surgical repair was performed using the Krackow suture technique, followed by structured postoperative rehabilitation. Despite favorable initial recovery, the patient experienced a traumatic open re-rupture of the tendon 14 weeks postoperatively during a sudden jumping motion. Emergency management involved wound closure and non-operative treatment of the incomplete re-rupture, including immobilization and gradual rehabilitation, enhanced with platelet-rich plasma (PRP) injected locally. The patient's subsequent recovery was favorable, allowing him to resume recreational sports six months post-treatment. This case underscores the importance of tailored rehabilitation combined with biological treatment (PRP) that enhances the healing process, the impact of modifiable risk factors, and the need for vigilance in post-repair activity to minimize re-rupture risk.

## Introduction

The Achilles tendon, the strongest and largest tendon in the human body, plays a crucial role in locomotion by transmitting forces generated by the calf muscles to the heel bone. Due to its high resistance, Achilles tendon is an option as an allograft even for ACL (anterior cruciate ligament) reconstruction [1]. Despite its strength, the Achilles tendon is prone to injury, particularly in active individuals engaging in high-impact or explosive activities. Acute ruptures of the Achilles tendon are common in sports-related injuries, and surgical repair is frequently employed to restore tendon continuity and functionality [2]. However, complications such as re-rupture, infection, and delayed healing remain a concern, particularly in patients with modifiable risk factors like smoking and elevated body mass index (BMI).

While re-rupture rates are generally low following surgical repair, such incidents can significantly impact functional outcomes and prolong recovery. Open re-ruptures of the Achilles tendon are rare and rarely described in the medical literature but pose unique challenges due to the potential for soft tissue compromise, infection, and tendon degeneration.

Although the incidence of Achilles tendon ruptures is approximately 31 cases per 100,000 individuals [2], this type of complication is infrequently observed. Typically, Achilles tendon ruptures occur approximately 6 cm proximal to the calcaneal insertion, potentially due to poor vascularization, which also hinders proper healing. These ruptures generally result from sports activities (particularly those involving jumping), falls from height, or stepping on uneven surfaces (e.g., potholes).

Several risk factors must be considered, such as age (more common between 30 and 40 years), sex (men are more prone to this condition), type of sport (e.g., football, basketball, tennis), local steroid injections, the use of certain antibiotics (e.g., fluoroquinolones), and obesity.

From a treatment perspective, there are two options: surgical and orthopedic management. Recently, there has been a decline in surgical treatments [3], as more studies and meta-analyses have demonstrated favorable outcomes in patients managed non-surgically. For instance, a multicenter study conducted in Norway, which followed over 500 patients for 12 months, found no significant differences in patient scores between those who underwent surgery (either open or minimally invasive) and those treated non-surgically [4]. However, the study reported a higher re-rupture rate in patients treated orthopedically. Other meta-analyses have reported re-rupture rates ranging from 2% to 12% [5].

PRP is a biological treatment, and it is used in many medical fields due to the high concentration of induction factors for tissue regeneration [6]. A study done to analyze the effects of PRP on Achilles tendon healing in rabbits has revealed that platelet-rich plasma shortened the inflammatory phase and promoted tendon healing during the proliferative phase [7]. A systematic review and meta-analysis revealed controversies of the efficiency of platelet-rich plasma injections for the treatment of acute Achilles tendon rupture [8].

There are few published articles detailing iterative ruptures of the Achilles tendon following surgical repair, as well as their management [9]. Optimal management strategies for these cases remain a topic of debate, as they depend on factors such as the extent of the re-injury, patient characteristics, and functional demands. In this article, we will present the case of a patient who underwent surgical treatment for an Achilles tendon rupture and returned to the clinic with a re-rupture of the same tendon.

## Case presentation

A 35-year-old male patient presented to the emergency department following an injury sustained during recreational football. He reported pain in the posterior region of the right ankle after feeling a snapping sensation in the area. The patient is a smoker with a BMI of 28.6 and no other significant medical history. At presentation, he was able to ambulate without support on the right lower limb and was accompanied by a caregiver.

Clinical examination revealed localized edema, bruising, and tenderness upon palpation of the Achilles tendon. The Thompson test was positive. Ultrasound evaluation showed a complete rupture of the Achilles tendon in its middle third.

After a thorough discussion with the patient, considering his age and level of daily physical activity, surgical intervention was planned and performed the following day. Open surgical repair was conducted, dissecting down to the paratenon and exposing the injury site. Careful debridement was performed, followed by a Krackow suture repair using non-absorbable multifilament Ethibond W4843 (Ethicon, Somerville, NJ, USA) thread, with knots tied while holding the foot in maximum plantar flexion. The paratenon and subcutaneous tissue were closed with non-absorbable 2-0 sutures, and the skin was closed with a monofilament thread.

Postoperatively, a below-knee equinus plaster splint was applied, and the patient was instructed not to bear weight on the affected limb for two weeks. At two weeks post-surgery, sutures were removed, and the wound showed favorable healing. The plaster splint was replaced with an AirWalker boot containing heel lifts to maintain the foot in equinus position. The heel lift height was reduced weekly, gradually decreasing the degree of plantar flexion.

The patient was advised to begin gentle ankle mobilization exercises. At six weeks, he started a structured physiotherapy program. At eight weeks, the patient transitioned to regular footwear while avoiding physical exertion.

**Figure 1:**
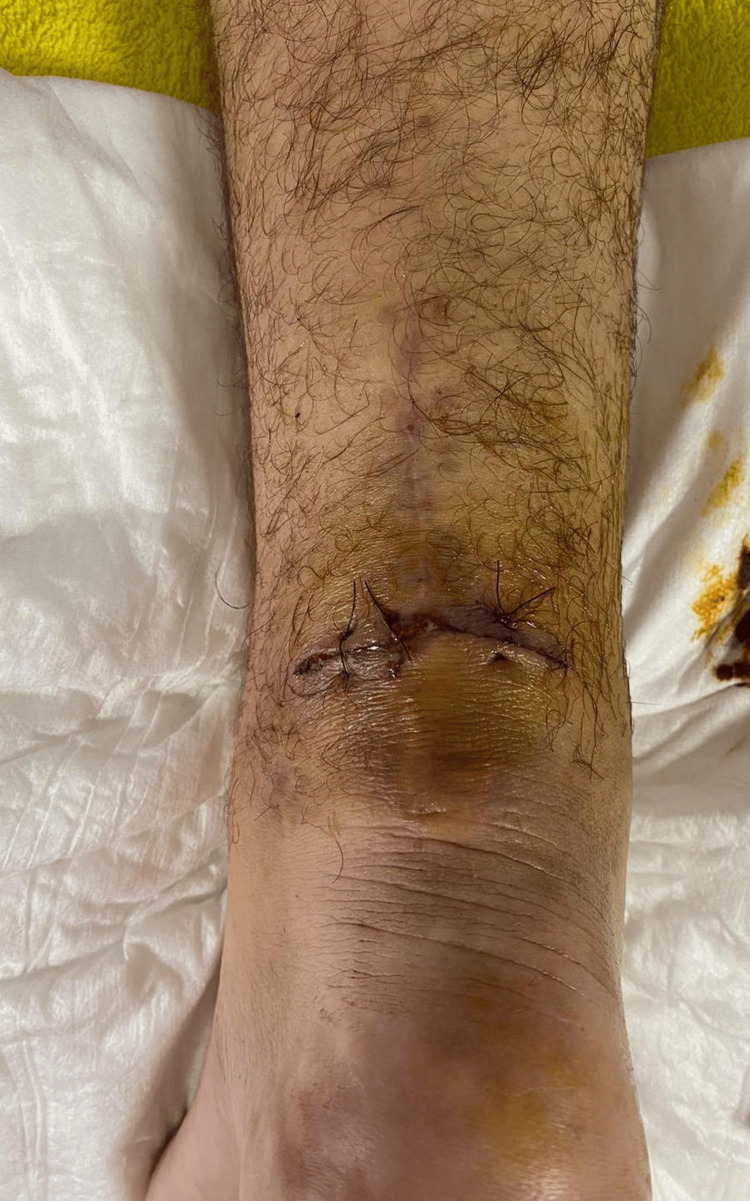
Wound sutured after re-rupture.

At 14 weeks postoperatively, while playing with his child, the patient performed a sudden jumping motion, experiencing severe pain in the same Achilles tendon, accompanied by bleeding from the site. In the emergency department, a 4 cm wound was noted, perpendicular to the prior surgical incision. The wound was sutured (Figure 1), and musculoskeletal ultrasound revealed a fortunately incomplete re-rupture of the Achilles tendon at the same level. Clinical examination confirmed a negative Thompson test, squeezing the calf initiated plantar flexion of the foot.

Based on clinical and imaging findings and after discussing options with the patient, orthopedic management was chosen. Initial immobilization with a plaster splint was followed by the Air-Walker boot. After two weeks in a plaster splint and another four weeks in the Air-Walker boot, the patient resumed physiotherapy.

The patient received three peritendinous injections with PRP under ultrasonography control. Our option for this type of treatment (PRP and three injections) was based on results revealed by other studies [10] and due to our experience with this product. Based on the benefits of PRP for acute Achilles tendon lesions, we tried to use the same approach for this open re-rupture. The first one was performed the first week from the recurrent rupture, the second one after another two weeks, and the third one after two weeks more.

The patient was informed regarding the risks and benefits of this minimally invasive procedure, and we had his written consent.

**Figure 2:**
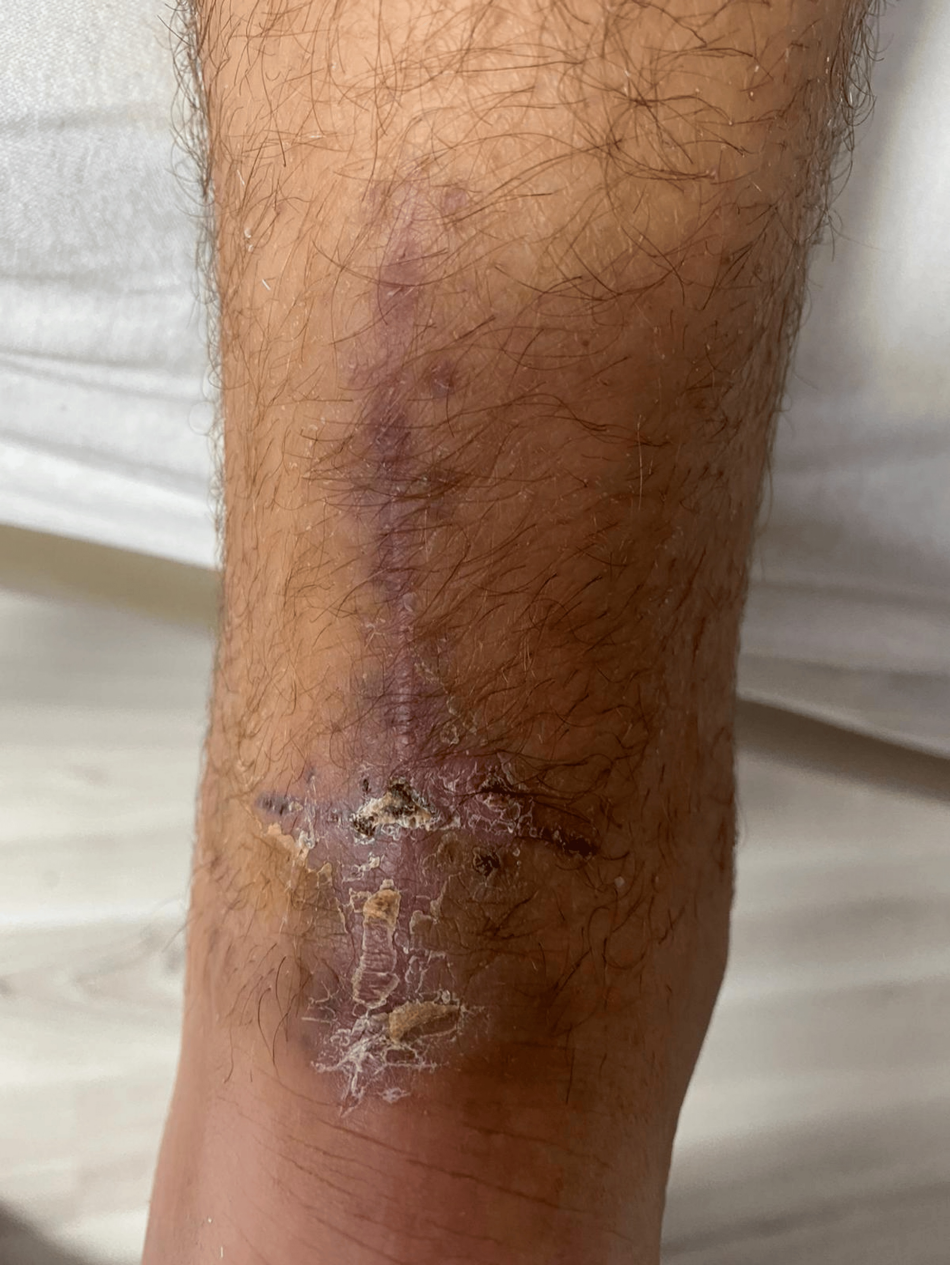
Wound healed, six weeks.

The subsequent recovery was favorable, both functionally and locally (Figure 2). The patient returned to recreational sports activities six months after completing the second treatment.

The patient demonstrated favorable recovery following conservative management of the re-rupture. At six weeks, he resumed physiotherapy, focusing on gentle mobilization and strength recovery. By six months, the patient had returned to recreational sports activities without significant pain or functional limitations. Clinical and ultrasonography examination at the final follow-up showed good tendon continuity, normal range of motion, and satisfactory functional outcomes.

## Discussion

The iterative rupture rate of surgically treated Achilles tendons varies significantly in the literature, with reported values ranging from 2% to as high as 12% [5]. Among these, cases of open re-rupture are extremely rare, and incomplete ruptures can be considered a distinct subcategory, being even less common. This case was described as an atypical scenario and to illustrate the feasibility of orthopedic treatment in such situations.

The rarity of these cases precludes statistical analysis and lends itself better to case report presentations. A number of authors have described one to two cases of open Achilles tendon re-ruptures following surgical treatment, with considerable variation in the management approaches employed. For instance, Hanada et al. reported two cases of open transverse re-rupture occurring at 4 and 13 weeks postoperatively, both of which showed favorable outcomes after surgical re-intervention [11]. Compared with his cases, ours was an incomplete rupture and benefited from a non-surgical treatment combined with biological therapy. 

Many authors have sought to identify the possible etiology underlying these cases [12]. Their conclusions suggest that adhesion formation between the repaired tendon and the subcutaneous tissue may exert axial traction on the skin, contributing to the phenomenon. Studies have shown that a faster and more aggressive functional recovery reduces the risk of re-ruptures [13].

Regarding the fact that we only had one special case of this type to treat and to report, we assume the limits: a reduced sample size and an evaluation limited to six months.

## Conclusions

The case described here is an atypical one, where the re-rupture was, fortunately, incomplete. This case highlights the challenges of managing Achilles tendon injuries, including re-rupture, and underscores the importance of patient education, new biological treatments (PRP), tailored rehabilitation, and consideration of individual risk factors for optimizing outcomes. Based on recent studies that revealed good functional outcomes in the long term after injecting PRP for Achilles tendon rupture, we adapted our treatment approach to the specific circumstances, opting for orthopedic management enhanced with PRP injections rather than surgical intervention, as is commonly described in the majority of Achilles tendon re-rupture cases. Given the rarity of these cases, the therapeutic approach requires further research. The same applies to analyzing the therapeutic utility of PRP in cases of recurrent Achilles tendon injuries.
